# A role for ceruloplasmin in the control of human glioblastoma cell responses to radiation

**DOI:** 10.1186/s12885-022-09808-6

**Published:** 2022-08-02

**Authors:** Charlotte Roy, Sylvie Avril, Claire Legendre, Bénédicte Lelièvre, Honorine Vellenriter, Sébastien Boni, Jérôme Cayon, Catherine Guillet, Yannick Guilloux, Michel Chérel, François Hindré, Emmanuel Garcion

**Affiliations:** 1 Université d’Angers, Inserm UMR 1307, CNRS UMR 6075, Nantes Université, CRCI2NA, F-49000 Angers, France; 2 Centre Régional de Pharmacovigilance, Laboratoire de Pharmacologie-Toxicologie, CHU Angers, 4 rue Larrey, F-49100 Angers, France; 3 Université d’Angers, SFR ICAT, Lentivec, F-49000 Angers, France; 4 Université d’Angers, SFR ICAT, PACeM, F-49000 Angers, France; 5 Nantes Université, Inserm UMR 1307, CNRS UMR 6075, Université d’Angers, CRCI2NA, F-44000 Nantes, France; 6 Université d’Angers, SFR ICAT, PRIMEX, F-49000 Angers, France; 7 GLIAD - Design and Application of Innovative Local Treatments in Glioblastoma, CRCI2NA, Team 5, Inserm UMR 1307, CNRS UMR 6075, Institut de Biologie en Santé (IBS) – CHU, 4 rue Larrey, Angers, France

**Keywords:** Glioblastoma, Radioresistance, Ceruloplasmin, Iron Metabolism, Hypoxia

## Abstract

**Background:**

Glioblastoma (GB) is the most common and most aggressive malignant brain tumor. In understanding its resistance to conventional treatments, iron metabolism and related pathways may represent a novel avenue. As for many cancer cells, GB cell growth is dependent on iron, which is tightly involved in red-ox reactions related to radiotherapy effectiveness. From new observations indicating an impact of RX radiations on the expression of ceruloplasmin (CP), an important regulator of iron metabolism, the aim of the present work was to study the functional effects of constitutive expression of CP within GB lines in response to beam radiation depending on the oxygen status (21% O_2_ versus 3% O_2_).

**Methods and results:**

After analysis of radiation responses (Hoechst staining, LDH release, Caspase 3 activation) in U251-MG and U87-MG human GB cell lines, described as radiosensitive and radioresistant respectively, the expression of 9 iron partners (TFR1, DMT1, FTH1, FTL, MFRN1, MFRN2, FXN, FPN1, CP) were tested by RTqPCR and western blots at 3 and 8 days following 4 Gy irradiation. Among those, only CP was significantly downregulated, both at transcript and protein levels in the two lines, with however, a weaker effect in the U87-MG, observable at 3% O_2_. To investigate specific role of CP in GB radioresistance, U251-MG and U87-MG cells were modified genetically to obtain CP depleted and overexpressing cells, respectively. Manipulation of CP expression in GB lines demonstrated impact both on cell survival and on activation of DNA repair/damage machinery (γH2AX); specifically high levels of CP led to increased production of reactive oxygen species, as shown by elevated levels of superoxide anion, SOD1 synthesis and cellular Fe2 + .

**Conclusions:**

Taken together, these in vitro results indicate for the first time that CP plays a positive role in the efficiency of radiotherapy on GB cells.

**Supplementary Information:**

The online version contains supplementary material available at 10.1186/s12885-022-09808-6.

## Introduction

Glioblastomas (GB), or grade IV astrocytomas, are the most deadly primary tumors of the central nervous system [1]. Despite an increase in the incidence of up to seven new cases per 100,000 habitants per year, therapeutic approaches have not significantly evolved over the last 30 years and remain palliative. Treatment generally consists of surgical resection when possible, followed by a combination of external beam radiation therapy with concomitant administration of the orally active alkylating agent temozolomide. Hence, the prognosis of GB remain very poor with a median survival of 14.6 months with radiotherapy amended with temozolomide versus 12.1 months with radiotherapy alone [2, 3].

To deal with this adverse clinical situation, partly due to resistance to ionizing radiation (IR), it is important to elaborate breakthrough therapeutics while exploring the development of new adjuvant treatments to improve conventional therapy for GB. In this context, targeting of iron signaling seems to be an interesting approach that has not yet been fully exploited.

Cancer cells require iron more than normal cells for proliferation [4] using it as a cofactor of ribonucleotide reductase involved in DNA biosynthesis and repair [5]. As such, depleting iron levels is a promising approach for anticancer therapy [6]. In GB, iron-related genes are dysregulated [7] and both TfR1 and TfR2 proteins are up regulated [8, 9]. Moreover, it has recently been demonstrated that cellular iron uptake and dependency is enhanced in GB stem-like cells (GBSC) [10]. In this regard, among markers associated with GBSC [11], AC133 / 1, which is an epitope present on the glycosylated protein CD133, is not only associated with radio-resistant phenotypes and with a negative clinical prognosis [12, 13] but also plays a key role in iron metabolism as validated by our team, with a first biological role identified for AC133 / 1 in the inhibition of transferrin cellular uptake [14]. In this context and like hypoxia which maintains the expression of AC133 / 1 [15] and supports the aggressiveness of GB [16], iron could be a key factor in the tumor ecosystem influencing the balance between sensitivity and resistance to treatment.

Iron chelation therapy has already had a significant clinical impact on treating iron-overload diseases or oxidative stress in neurodegenerative diseases [6, 17]. Deferasirox, which has high iron chelating ability, showed antineoplastic properties in numerous human cancer cells and in preclinical studies [18]. However, the complexity of using deferasirox in GB treatment has to be highlighted due to tumor heterogeneity and impaired activity in the context of low oxygen tension [19]. Important hypoxic zones are common to many cancers and GB [20] and are partly responsible for the resistance of tumors to radiotherapy [21]. To eradicate the tumor, IR acts in two ways: i) either directly by causing damage to the DNA resulting in cell death, or ii) indirectly via reactive oxygen species formation (ROS) that will induce DNA damage and finally cell death. Under normoxic conditions, ROS is effective at promoting DNA damage but under hypoxic conditions, oxygen limitation limits ROS production and therefore reduces the effectiveness of radiotherapy [22]. In addition to oxygen, other factors are known to be central for ROS production with iron arguably one of the most pivotal. By acting as co-factor, iron could strongly influence the efficiency of radiotherapy [23]. In addition to being a proliferation and cell growth promoter, iron takes part, with oxygen, in Fenton’s reaction to produce ROS. Indeed, free iron (non-bound) is easily oxidized and can stimulate ROS production, so, excess iron is toxic [24]. In this manner iron could trigger cancer initiation through increasing genetic alterations by ROS production but also could participate, as an anti-cancer agent, by triggering elevated ROS production to induce cell death following radiotherapy or by lipid peroxidation, a cell death process called ferroptosis [25].

Since iron could play a central role in tumor growth and also be beneficial to radiation efficiency, the present work has been carried out to investigate and decipher the biological effect of IR on iron partners in GB cells and the role that in turn these could have on the effective molecular and cellular responses to radiation. Two human glioblastoma cell lines, U87-MG and U251-MG cells were used and exposed to beam radiation (X-ray) before being analyzed for levels of cell proliferation, cytotoxicity, apoptosis and iron metabolism-related gene profile under two oxygenation conditions: experimental normoxia (21% oxygen) and physioxy (3% oxygen) [20]. Results showed that among iron partners investigated, only ceruloplasmin (CP) was regulated and strongly decreased at day 8 following an external beam radiation in both cell lines. As first identified by Holmberg and Laurell, CP is the major copper-carrying protein in the blood [26]. CP is expressed in both a secreted and GPI-anchored form and exerts broad functions ranging from iron and copper homeostasis, oxidation of organic amines, to anti and pro-oxidant activities [27]. Interestingly, through its ferroxidase activity, it is also involved in iron metabolism converting ferrous iron Fe^2+^ to ferric iron Fe^3+ ^[28] which is internalized by cells via transferrin (Tf) [29]. Cellular marker of stress caused by radiation or factor of radio-sensitization, the observed decrease of CP in GB cells following IR allowed us to ask the new question of its potential role in the effectiveness of radiotherapy. Here, a more specific study using genetically modified GB cells to overexpress or under-express this protein has been carried out to investigate this hypothesis.

## Materials and methods

### Cell culture

Human GB U87-MG cells (ATCC® HTB-14™) were purchased from the American type Culture Collection (ATCC, LGC Standards, Molsheim, France) while U251-MG cells were a gift from C. Griguer and were originally obtained from Dr. D.D. Bigner (Duke University, Durham, NC). Human U251-MG and U87-MG cells are routinely cultured in Dulbecco's modified Eagle's medium (DMEM) containing 4.5 g/L glucose and L-glutamine (Lonza) supplemented with 10% (v/v) fetal bovine serum (FBS) (Lonza) and a combination of 100 units/ml penicillin and 100 μg/ml streptomycin. Cells were maintained at 37 °C in a humidified 5% CO_2_ atmosphere with 21% or 3% oxygen.

### Lentiviral infection to generate U251-MG with constitutive shRNA CP and U87-MG with ORF cDNA CP

To obtain U87-MG cells overexpressing CP (CP +  + cells), these cells were infected with a lentifect purified lentiviral particles ready to use from Tebu-Bio. These particles contained gene synthesis ORF expression clone for XM_006713499.1 (3273 bp), coding for CP, in a pReceiver-Lv105 vector with CMV as promotor and puromycin as a selection marker. A negative control lentiviral particles purified was also used as “scrambled” (Control CP + +). To obtain U251-MG cells depleted CP (CP shRNA cells), these cells were infected with a lentiviral particle containing CP shRNA cDNA clone (NM_000096) in a plasmid PLKO.1 CMV-NEO ready to use from Sigma. An empty plasmid was used for negative control (Control CP shRNA).

### Ionizing radiation procedure

10,000 cells/cm2 were seeded for all experiments except for western blotting with 15,000 cells/cm2 and MitoSOX experiments with 20,000 cells/cm2. Prior to irradiation, medium was removed and replaced 8 or 24 h after splitting by DMEM medium with antibiotics and with N1 medium supplement (Sigma). Irradiation was performed with the CP-160 cabinet x-ray system (Faxitron, Edimex, Le Plessis Grammoire, Angers, France) which delivers a dose of 1.5 grays (Gy) a minute. Irradiation was continued for 2.66 or 10.66 min in order to reach a dose of 4 or 16 Gy, respectively. The cells were covered during process.

### Cell viability assay

Three days after irradiation, cells were washed with PBS 1 × and fixed in 95% ethanol / 5% acetic acid (v/v) for 20 min at 4 °C. Hoechst 33,342 used at 1.5 μg/mL in PBS 1 × was incubated for 30 min. For each condition, 10 fields were counted using a fluorescent microscope (Axiovert 40 CFL Zeiss, Marly le Roi, France) and the number of nuclei were determined. Two days after irradiation, the media was replaced with combined MTS/PMS solution (CellTiter 96® AQ_ueous_ non-radioactive cell proliferation assay, Promega) and incubated 2 h and 30 min at 37 °C in a humidified 5% CO_2_ atmosphere with 3% oxygen. The absorbance of the media was measured at 490 nm using LabSystems Multiskan.

### Cytotoxicity assay

Three days after irradiation, the release of lactate dehydrogenase (LDH) into cell culture supernatants was measured using LDH cytotoxicity detection kit (Roche Diagnostics) according to the manufacturer's instructions. GB cells treated with Triton X-100 at 0.1% (v/v) were used as positive control of cytotoxicity and assigned the arbitrary value of 100%.

### Caspase-3 activity

Three days after irradiation, total proteins were isolated from GB cells by sonication in a lysis buffer (20 mM PIPES pH 7.2, 100 mM NaCl, 1 mM EDTA, 0.1% KCl w/v, 10% sucrose w/v, DTT 10 mM and PMSF 100 μM). Proteins (30 µg) were incubated at 37 °C with 80 μM N-acetyl-Asp-Glu-Val-Asp-7-amino-4-methylcoumarin (N-acetyl-DEVD-AMC) and the kinetics of caspase activity was measured with a Fluoroskan Ascent FL (Thermofisher scientific, Illkirch, France) at the excitation/emission wavelength pair of 380/440 nm.

### Flow cytometry

All experiments were performed using MACSQuant® (Miltenyi) automate flow cytometer.

#### CP expression

To validate generated clones, CP expression was measured by flow cytometry using polyclonal antibody (Ab) anti-CP (Dako) with secondary Ab Goat F(ab’)2 Anti-Rabbit IgG Fc FITC (ab6018, Abcam). Secondary Ab alone was considered as isotype control. Results are expressed as relative fluorescence intensities (RFI) as follows: (A/B), where A and B were the Mean Fluorescence Intensity (MFI) values obtained with specific and isotype control Abs, respectively.

#### Apoptosis

Early and late apoptosis were assessed 24 h after IR by studying the expression of annexin-V and propidium iodide. Results are expressed as MFI. 2. 

#### Anion superoxide

One day after IR and after HBSS washed, GB cells were incubated with MitoSOX™ Fluorescent (2 µM) in a plate for 15 min at 37 °C in the dark. After twice HBSS washed and 5 min before analyzing in the cytometer, 1 µM of Sytox Blue, to analyzed live cells, was added.

### ICP-MS method

Two or 48 h after IR, the supernatant or cells, to evaluated extracellular and intracellular metals respectively, were incubated with 0,1 g/l of EDTA or not for 10 min in room temperature and then frozen until experiment. Solutions incubated with EDTA allow to measure free iron or copper only while solutions alone allow to measure total iron or copper; then bound metals has been determined as follow: total metals – free metals. After 1/20 dilution of samples using 0.5% nitric acid solution containing internal standards (Ge, Rh) the preparation was analyzed using Inductively Coupled Plasma Mass Spectrometry (ICP-MS) 7800 (Agilent). To avoid any interference, helium was used as collision gaz (flow rate of 3.8 ml/min). The calibration ranged from 2.5 to 800 µg/L.

### Western blot analysis

Total proteins were isolated from GB cells by sonication in a lysis buffer composed of 50 mM HEPES, pH 7.5, 150 mM NaCl, 1 mM EDTA, pH 8, 2.5 mM EGTA, pH 7.4, 0.1% Tween 20, 10% glycerol, 0.1 mM sodium orthovanadate, 1 mM sodium fluoride, 10 mM glycerophosphate and 0.1 mM phenylmethylsulfonyl fluoride (PMSF). Proteins (20 – 50 µg) were resolved on 4–20% Mini-PROTEAN® TGX™ precast polyacrylamide gels (Bio-rad) or 15% polyacrylamide gels and transferred to an Amersham GE Healthcare nitrocellulose membrane (0.45 μm pore size) (Fisher scientific). The following antibodies were used according to the manufacturer’s instructions: a mouse anti-human CP at dilution 1:1000 (3B11, Fisher scientific), a mouse anti-human γH2AX, recognizing phosphorylation of the Ser-139 residue of the histone variant H2AX, at dilution 1:1000 (ab26350, Abcam), a rabbit anti-human Superoxide Dismutase 1 at dilution 1:2000 (SOD1, Abcam). An anti-human Mouse Heat Shock Cognate Protein 70 (HSC70) at dilution 1:10,000 (sc-7298, B-6, Santa Cruz) or mouse anti-human β-actin at dilution 1:4000 (AC-74, Sigma) were used as a loading control. Goat anti-Mouse or anti-Rabbit IgG Secondary Antibody, HRP conjugate (Fisher scientific) were used at a dilution of 1:10,000. Detection was performed on SuperSignal™ West Femto Maximum Sensitivity Substrate (Fisher scientific) with a ChemiCapt 3000 imaging system (Vilber Lourmat, Marne-la-Vallée France).

### RNA isolation, cDNA synthesis and quantitative real-time PCR

Total RNAs were extracted from U251-MG and U87-MG cells using the RNeasy Mini Kit (Qiagen). 1 µg of RNA was reverse-transcribed into complementary DNA using random primers and Superscript II according to the manufacturer’s recommendations (Invitrogen, Life Technologies, Thermofisher scientific). Quantitative real-time PCR was performed using the Sybrgreen Master Mix (Thermofisher Scientific) and the LightCycler 480 (Roche Diagnostics, Meylan, France) according to the manufacturer’s instructions. Primer pairs used for each transcript were specifically designed using Primer Blast software. The relative abundance of mRNA levels was calculated with the 2^−ΔΔCt^ method for each gene using as reference genes Ct values of Hypoxanthine–guanine PhosphoRibosylTransferase 1 (HPRT1) and Tyrosine 3-Monooxygenase/Tryptophan 5-Monooxygenase Activation Protein Zeta (YWHAZ) for normalization. Designed CP primers can amplify and recognize cDNA for both CP and GPI-CP or specifically the integration of the nucleic acid coding for the 30 amino acid peptide (GPI-CP only). A such, Total CP represent the addition of GPI-CP and none-GPI-CP. Sequences of primer pairs are represented in Table 1.

**Table 1:**
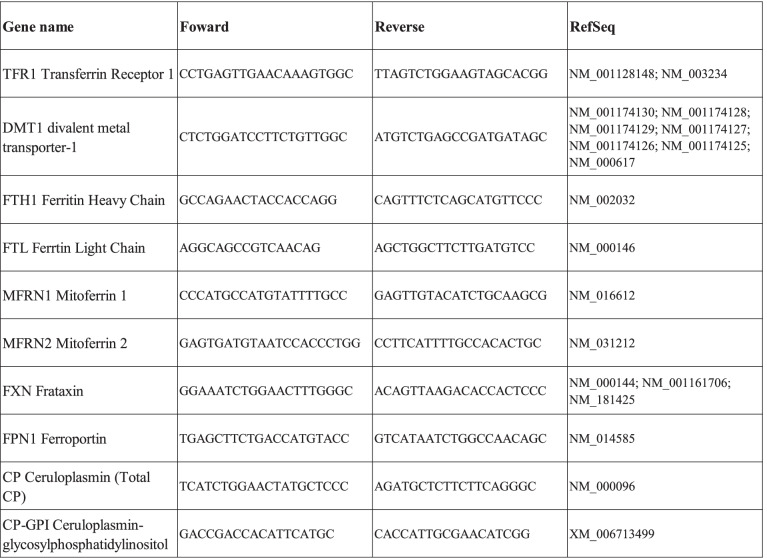
Sequences of primer pairs

The table above shows the forward and reverse sequences for all primers used in the manuscript. Designed CP primers can amplify and recognize cDNA for both CP and GPI-CP or specifically the integration of the nucleic acid coding for the 30 amino acid peptide (GPI-CP only). As such, total CP represent the addition of GPI-CP and none-GPI-CP

### Comet assay

Two hours after IR, GB cells were washed with PBS 1X and incubated 15 min in Trypsin 0.005% at 37 °C. After centrifugation at 1500 rpm for 5 min at 4 °C, cold PBS 1X was added on cell pellet which then was kept in ice and dark. The following steps are performed under the same conditions. 1/10 cells mixed with low gelling Agarose 1% (Type VII, Sigma) maintained at 37 °C was rapidly deposited on slides pre-coated with routine agarose 1% (Sigma) to obtain 2500 cells per slide and then covered with coverslip. After 10 min, coverslip has been removed and slides were incubated in cold lysis buffer (NaCl 2.5 M, Na_2_EDTA 100 mM, Tris–HCL 10 mM and freshly added Triton x-100 at pH 10) overnight. After incubation for 20 min in cold electrophoresis buffer (NaOH 0.03 M, Na_2_EDTA 2 mM at pH 12.3), slides are placed in an electrophoresis chamber, put on ice and containing this same buffer, and set to migrate 30 min at 0.74 V/cm and 20–30 mA. Slides were neutralized in Tris–HCL 0.4 M at pH 7.5 and room temperature for 20 min and then washed once in distilled water. DNA was marked by Sybr Green (1/50 000, ThermoScientific) for 20 min and then washed once in distilled water. 20 pictures per condition were taken using epifluorescence microscope.

### Statistical analysis

Three or more independent biological replicates were performed for all experiments described in this manuscript. Data are represented as mean ± SEM. Statistical analyses were performed using Graphpad PRISM (La Jolla, CA, USA). Multiple groups were compared by using a one-way analysis of variance (ANOVA) or two-way ANOVA when cell populations have been compared. Two groups were compared by using an unpaired Student’s t test (two-tailed); *p*-values of < 0.05 were considered statistically significant.

## Results

### Differential efficiency of ionizing radiations depending on the cellular context: the case of U87-MG and U251-MG cells

**Fig. 1:**
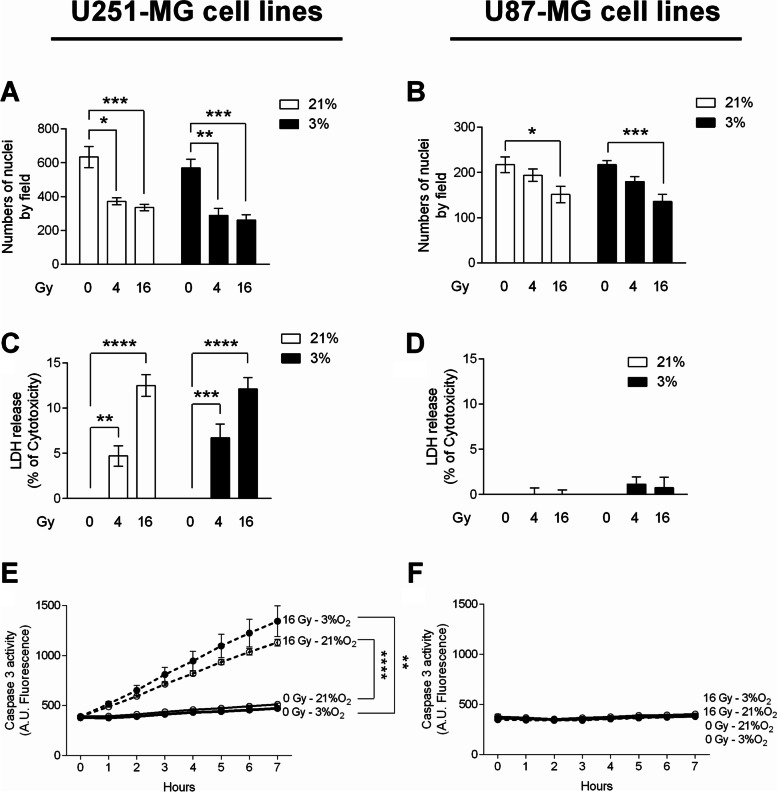
Ionizing radiation inhibits proliferation linked with increased cytotoxicity and apoptosis but only in U251-MG GB cells. For these experiments, GB cells were cultivated at 21% or 3% oxygen in non-irradiated condition (0) or 3 days after an irradiation of 4 or 16 Gy. Numbers of nuclei of U251-MG (**A**) and U87-MG (**B**) GB cells are expressed as mean ± Standard Error of the Mean (S.E.M.) (*n* = 3). Measure of Lactate Dehydrogenase (LDH) release into cell culture medium of U251-MG (**C**) and U87-MG (**D**) GB was assessed. Cytotoxicity is expressed as mean percentage ± SEM (*n* = 3) of the total amount of LDH released from cells and relative to GB cells treated 0.1% Triton X-100, given the arbitrary percentage of 100. DEVD-AMC caspase-3 activity in U251-MG (**E**) and U87-MG (**F**) GB cells is expressed as mean arbitrary units (A.U.) of fluorescence per 30 µg of proteins ± SEM (*n* = 3). One-way ANOVA test was performed between non-irradiated (0 Gy) or irradiated (4 or 16 Gy) conditions (^*^, *p*-value ≤ 0.05; ^**^, *p*-value ≤ 0.01; ^***^, *p*-value ≤ 0.001; ^****^, *p*-value ≤ 0.0001)

In order to further establish a possible rationale between the impact of irradiation and the cellular metabolism of iron, the response to external beam X-ray of two human GB cell lines, U251-MG and U87-MG, were firstly characterized in terms of proliferation, cytotoxicity and apoptosis. As hypoxia is important feature in the GB biology, those effects were studied both at 21% (experimental normoxia) and 3% oxygen (physioxia) (Fig. 1).

Three days after radiation of 4 or 16 Gy, a significant decrease in proliferation was observed in U251-MG cells (Fig. 1A) in each condition of oxygenation. Moreover, the impact of IR on U251-MG cells was fully correlated with the induction of LDH release (Fig. 1C) and caspase-3 activity (Fig. 1E). This observation suggests that the antiproliferative effect of X-rays is probably mediated by cell death, including necrosis and apoptosis. In contrast, IR had no significant effect on U87-MG cells in the tested conditions (Fig. 1D and F) with even so a significant decrease in numbers of nuclei for 16 Gy-irradiation in both oxygenation condition (Fig. 1B). As a result, U251-MG cells appear more radiosensitive than U87-MG cells.

These results are consistent with previous work on genetic status showing that mutant p53 cells are generally more sensitive to ionizing radiations than wild-type p53 cells [30]. Hence, U251-MG are PTEN-/p53-mutated cells while U87-MG are PTEN-mutated and p53 wild-type cells. Although radio-sensitiveness can also be explained by the fact that U251-MG cells have less DNA damage repair activity of Ape1 than U87-MG cells [31], the contribution of constituent elements of the tumor ecosystem, affecting the impact of radiations, warrants further investigation; this is the case of iron and of the cellular partners of its metabolism.

### Ionizing radiation modulates the expression of iron metabolism-related genes and preeminently CP

**Fig. 2:**
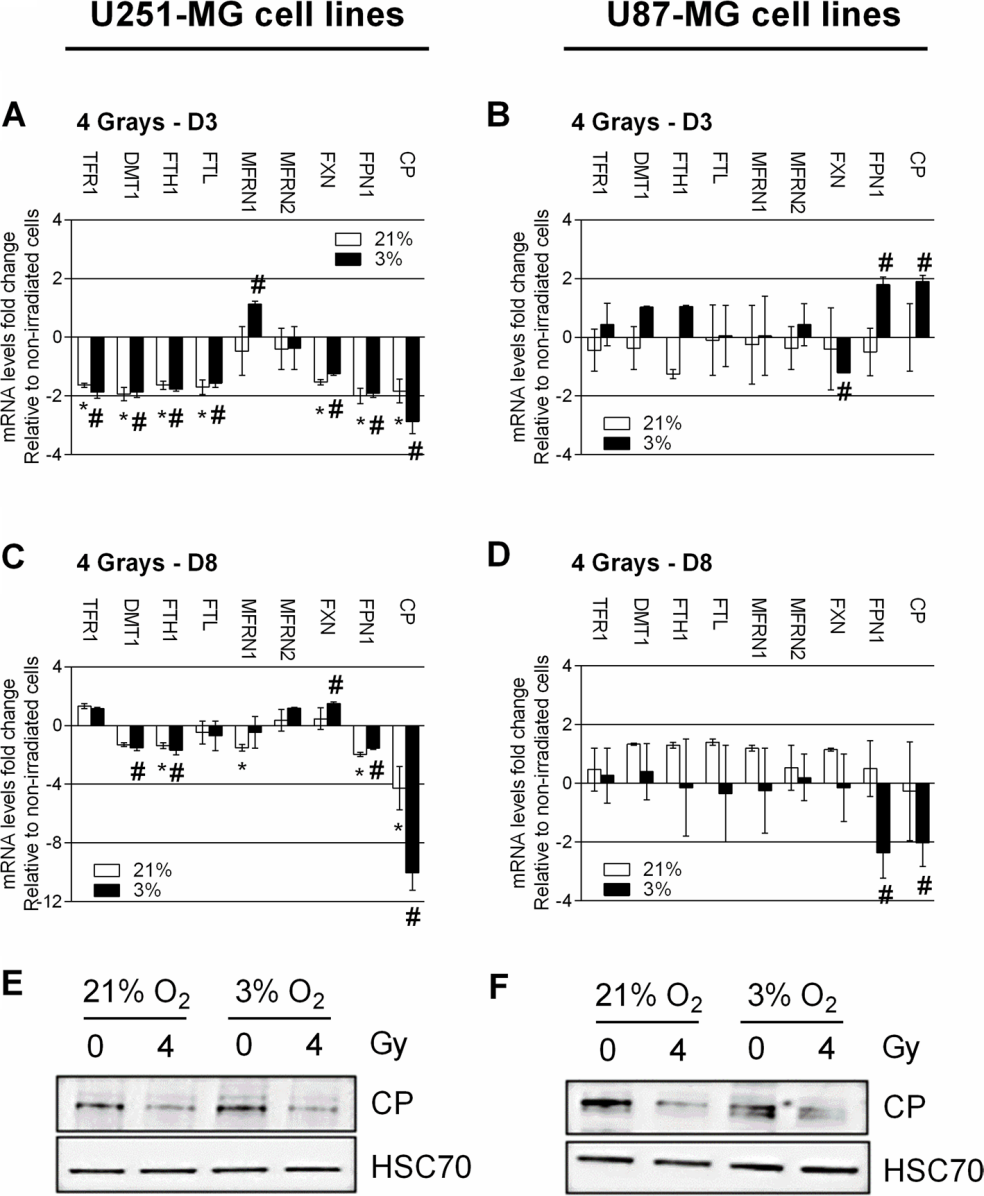
Change in expression of iron metabolism-related genes and ceruloplasmin protein in response to IR in U251-MG and U87-MG cells. mRNA level fold change of transferrin receptor 1 (TFR1), divalent metal transporter 1 (DMT1), heavy and light ferritin chain (respectively FTH1 and FTL), Mitoferrin 1 and 2 (respectively MFRN1 and MFRN2), frataxin (FXN), ferroportin (FPN1) and CP in U251-MG (**A** and **C**) and U87-MG (**B** and **D**) GB cells cultivated at 21% or 3% oxygen at 3- (**A** and **B**) or 8- days (**C** and **D**) post-IR with 4 Gy. Fold change are expressed as mean ± SEM (*n* = 3) and relative to non-irradiated control condition, given the arbitrary value of 1. Multiple T-Test was performed (^*^, *p*-value ≤ 0.05 for 21%; # *p*-value ≤ 0.05 for 3%). Levels of CP protein and heat shock cognate protein 70 (HSC70) protein used as loading control protein in U251-MG (**E**) and U87-MG (**F**) GB cells cultivated at 21% or 3% oxygen in non-irradiated condition (0) or 3 days after an irradiation of 4 Gy. Western blot data represent one of three independent experiments with comparable results

Starting with the idea that gene modulation is rarely pointless but linked to specific functions, the impact of external beam radiation (IR) on the expression of iron metabolism-related genes was studied in the two cell lines U87-MG and U251-MG. Cells were exposed to 4 Gy for 3 days to follow direct impact of irradiation on iron metabolism-related genes in treated cells or for 8 days to study the iron-related transcriptomic characteristics of the survival cell fraction. mRNA level provides a measure of gene expression and also of the stability of mRNAs, which can be subjected to IRP/IRE regulation but also RNA interference (miRNA). Hence, the expression of transcript involved in (i) iron uptake with the Tf receptor 1 (TFR1) and divalent metal transporter-1 (DMT1), (ii) iron storage with the two ferritins, heavy (FTH1) and light (FTL) chain, (iii) iron mitochondria metabolism with the two mitoferrin (MFRN1 and 2) and frataxin (FXN) and iron efflux with ferroportin (FPN1) and total CP (CP) were studied (Fig. 2).

In U251-MG cells, 3 days after 4 Gy irradiation, a global repression of iron metabolism-related genes was observed except for the MFRN1 at 3% oxygen. However, for all genes studied, the fold-change is less than 2 except for CP at 3% of oxygen (Fig. 2A). At 8 days, the profile is slightly different as TFR1, MFRN2 and FXN are up-regulated whereas all the other transcripts are down-regulated. However, the fold-change is less than 2 except for CP, where at 21%, a down-regulation of fourfold is observed and at 3%, a down-regulation of tenfold is observed (Fig. 2C). For U87-MG cells, which is a relatively radio-resistant cell line, modulation of gene expression following 4 Gy irradiation at 3 and 8 days, is less than a fold-change of 2 for all genes studied (Fig. 2B, D). Nevertheless, a significant decrease of CP is observed at 8 days and at 3% of oxygen (Fig. 2D). In order to confirm the downregulation of CP expression by 4 Gy irradiation at 8 days, protein levels were examined. In both cell lines, U251-MG and U87-MG (Fig. 2E and F, respectively), western blotting indicated a downregulation of CP protein expression after an irradiation of 4 Gy both at 21% and 3% oxygen with similar expression at both O_2_ levels.

Because CP was found to be highly expressed at the surface of U251-MG GB cells but only weakly expressed at the surface of U87-MG GB cells (data not shown), U251-MG and U87-MG were transduced for specific CP short hairpin (sh)RNA-knockdown and stable CP overexpression, respectively. Analyses of response to treatment were then performed at 3% O_2_, the physioxic condition in which regulation of CP by irradiation was most prominent.

### CP knockdown and CP overexpression in U251-MG and U87-MG cells, respectively

To evaluate the role of CP in GB cell lines after IR, U251-MG cells were transfected by a CP shRNA while U87-MG cells were transduced with a CP cDNA to obtain a specific shRNA-mediated CP knockdown (named CP shRNA cells) and a cDNA-mediated CP-overexpression (named CP +  + cells), respectively.

**Fig. 3:**
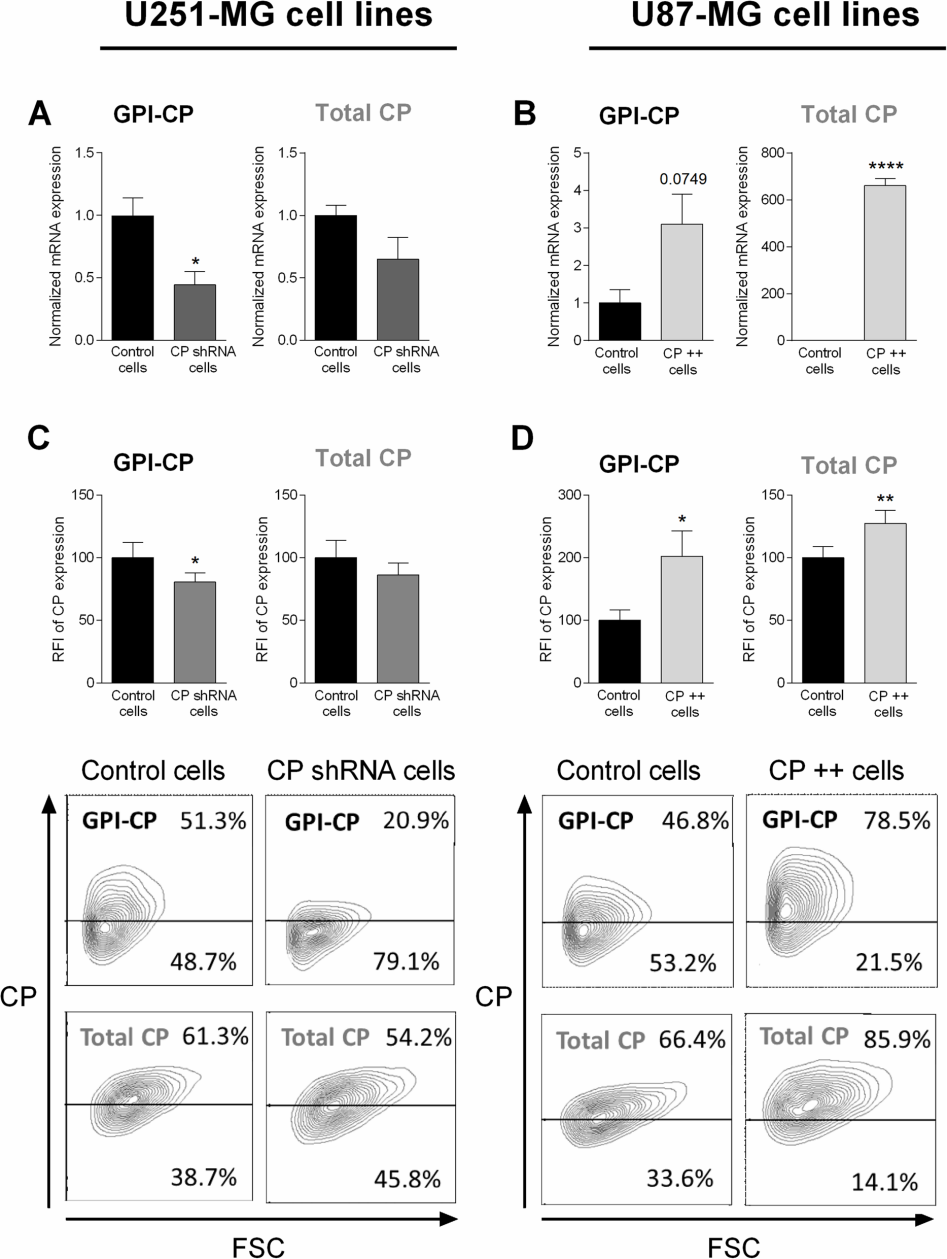
Identification of stable knockdown or overexpressing of CP in GB cells. U251-MG and U87-MG GB cell lines were respectively transfected with CP short hairpin (sh)RNA and CP cDNA. To validated clone construction, CP gene expression level was measured by quantitative real-time PCR in stable transfected U251-MG (**A**) and U87-MG cells (**B**). GPI-CP expression (membrane-bound CP) is represented in left, while CP all isoforms expression is represented in right (**A** and **B**). CP expression is expressed in mRNA expression normalized to their own control ± SEM (*n* = 3). CP protein level was measured by flow cytometry in stable transfected U251-MG (**C**) and U87-MG cells (**D**). GPI-CP expression (membrane-bound CP), obtained without permeabilization, is represented in left graph, while CP all isoforms expression, obtained after permeabilization, is represented in right graph (**C** and **D**). CP expression is expressed as RFI ± SEM (*n* = 5). T-test was performed between normalized Control versus Clones conditions (^*^, *p*-value ≤ 0.05; ^**^, *p*-value ≤ 0.01; ^****^, *p*-value ≤ 0.0001)

In order to quantify the nature of the modification of the transfected cells, CP gene and protein expression were respectively measured in U251-MG and U87-MG cell lines cells by quantitative real-time PCR and flow cytometry following transfection (Fig. 3). For each panel, the left graph corresponds to the CP anchored to the membrane by a glycosylphosphatidylinositol (GPI-CP) and the one on the right to the total CP. Specific primers of the two forms were used for RT-qPCR while the cells were permeabilized or not to obtain total CP and GPI-CP, respectively, in flow cytometry. In U251-MG cells, shRNA transfection efficiently decreased CP production (CP shRNA cells) compared to cells transfected with irrelevant shRNA (Control cells) (Fig. 3A, C). Results were significant for GPI-CP thoughless obvious for the total CP (left vs. right). Concerning U87-MG cell lines, cDNA transfection increased CP production and transcript (CP +  + cells) compared with cells transfected with the empty vector (Control cells) (Fig. 3B and D). Interestingly, the molecular impact of transfection at mRNA levels (down- or up-regulation) were less pronounced when looking at protein levels, thus suggesting multiple post-transcriptional regulation of CP expression. Moreover, the shRNA cDNA clone (NM_000096) used in this study for U251-MG cell lines targets the membrane CP isoform (GPI-CP) which would explain a more pronounced downregulation for this CP isoform. In contrast, the ORF cDNA used for overexpression of CP in U87-MG GB cells includes both isoforms of CP (anchored and secreted). Note however, as previously described in the brain [32] secreted CP is absent in these GB cell cultures as indicated by ELISA in supernatants (data not shown).

**Fig. 4:**
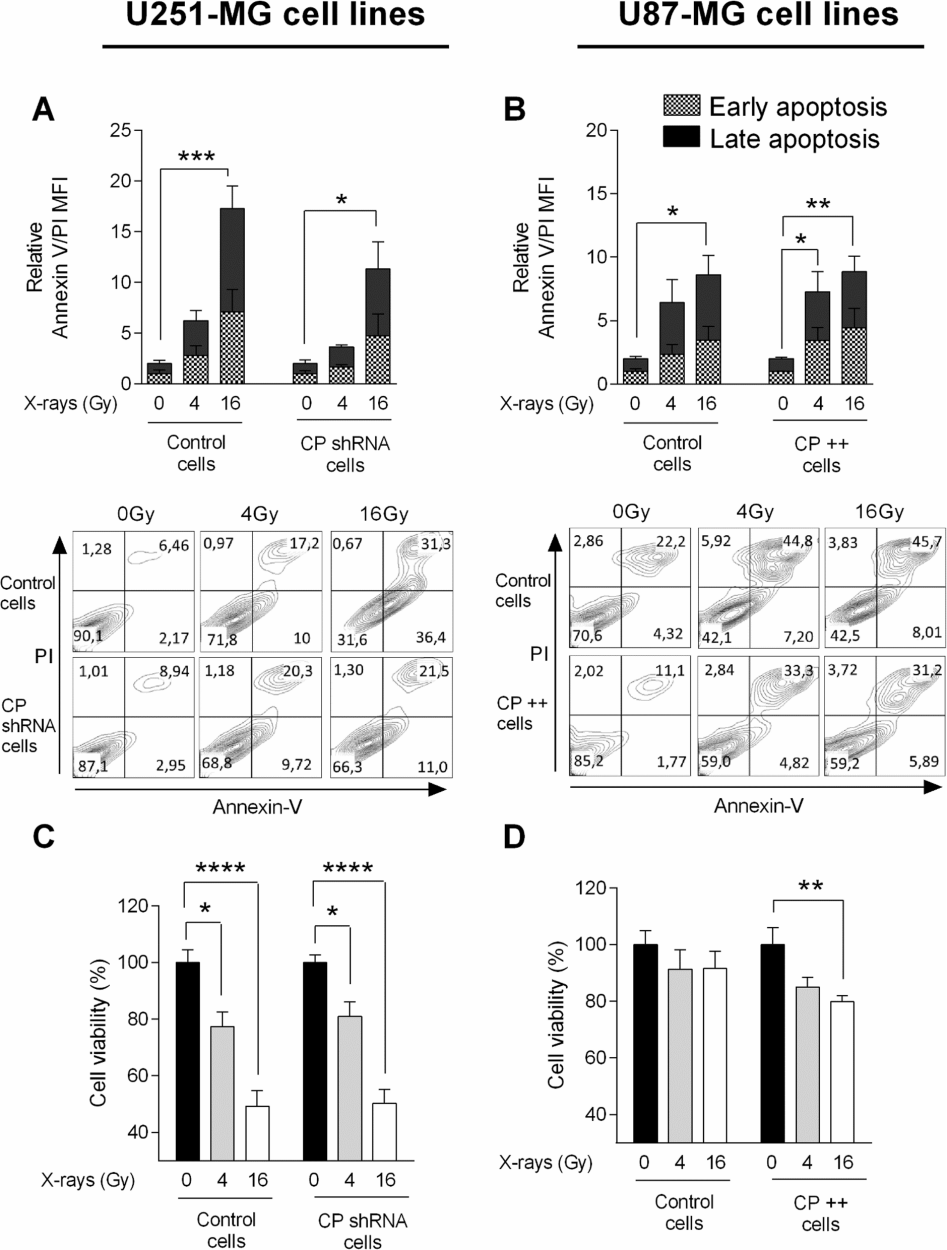
Modulation of cellular viability and apoptosis in CP-modulated cells post-IR. Early and late apoptosis was evaluated by measure of annexin V positive cells and Ann V and IP positive cells, respectively, by flow cytometry in stable transfected U251-MG (**A**) and U87-MG GB (**B**) cell lines 24 h after a single exposure to 4 Gy- or 16 Gy-irradiation at 3% oxygen. Apoptosis is expressed as relative MFI of annexin V and PI ± SEM (*n* = 3). In same conditions but here 48 h after IR, MTS viability assay was performed in U251-MG (**C**) and U87-MG cells (**D**). MTS viability is expressed as DO ± SEM (*n* = 7). One-way ANOVA with multiple comparisons (each group was compared to their own normalized control) was performed between Control vs. irradiated cells (^*^, *p*-value ≤ 0.05; ^**^, *p*-value ≤ 0.01; ^***^, *p*-value ≤ 0.001; ^****^, *p*-value ≤ 0.0001)

In order to evaluate the impact of CP in terms of cell behavior and damage, after treatment in pathophysiological conditions, stable CP transfected cells were either irradiated or not in hypoxic condition (3% O_2_) and compared to their own control cells (naked vector or scramble shRNA transfected). Note that from Fig. 4, we analyzed the cell populations independently (although the graphs are grouped). For all parameters evaluated in this study, as exemplified by viability, apoptosis, oxidative stress and DNA damage, it is important to note that no difference was observed at basal level (without radiations) in CP +  + or CP shRNA cells as compared to their matched control (data not shown); apart from SOD1 expression in U87-MG cell lines, specified in the manuscript.

### Impact of CP depletion and overexpression on GB cell survival following ionizing radiation

The consequence of CP depletion and overexpression on the survival of GB cells was explored after a single exposure to 4 Gy- or 16 Gy-irradiation. For this purpose, early and late apoptosis was investigated through exploration of the expression of annexin-V and propidium iodide 24 h after radiation exposure while viability was tested by MTS assay at 48 h (Fig. 4). Although the effects of irradiation are clearly visible as shown by inducing annexin V/propidium iodide labeling in the two cell types in Fig. 4A and B, it should be noted that the dose dependence (0, 4, 16 Gy) presents a specific profile of each cell type. ShRNA knockdown of CP in the U251-MG cell line results in a decrease in this apoptosis visible at 24 h (Fig. 4A). Indeed, we observed a 17-fold increase of apoptosis for U251-MG Control cells and only a 11-fold increase for CP shRNA cells at 16 Gy of irradiation (for 4 Gy, sevenfold increase versus threefold increase respectively). In contrast, the relative overproduction of CP in the U87-MG line does not impact annexin V/propidium iodide labeling at 24 h (Fig. 4B). By focusing on cell viability, the effectiveness of radiotherapy was clearly visible on each cell type (Fig. 4C and D). While U251 cells are clearly radiosensitive with and without manipulation of endogenous levels of CP (control cells and CP shRNA cells, Fig. 4C), commonly radioresistant U87-MG cells exhibit a significant increased sensitivity to irradiation at 16 Gy and to a lesser extent at 4 Gy when CP is overexpressed (CP +  + cells vs. Control cells, Fig. 4D).

### Impact of CP depletion and overexpression on oxidative stress following ionizing radiation: superoxide anion production and SOD1 expression

Any differences observed (the reduction of apoptosis by reducing CP in U251-MG or the decrease in viability by increasing CP in U87-MG) support a role for CP in radiation sensitivity that goes beyond a pointless expression leading to an impact on prevention or repair of radiation damages. Having established on the one hand that GB tumor cells regulate their level of CP in response to radiations and on the other hand that CP is involved in radio-sensitivity of GB cells, the mechanisms at the origin of this involvement were investigated on the basis of a possible CP role on oxidative stress and levels of reactive oxygen species (ROS) that may be present in GB cells and on the DNA damage/repair activity.

**Fig. 5:**
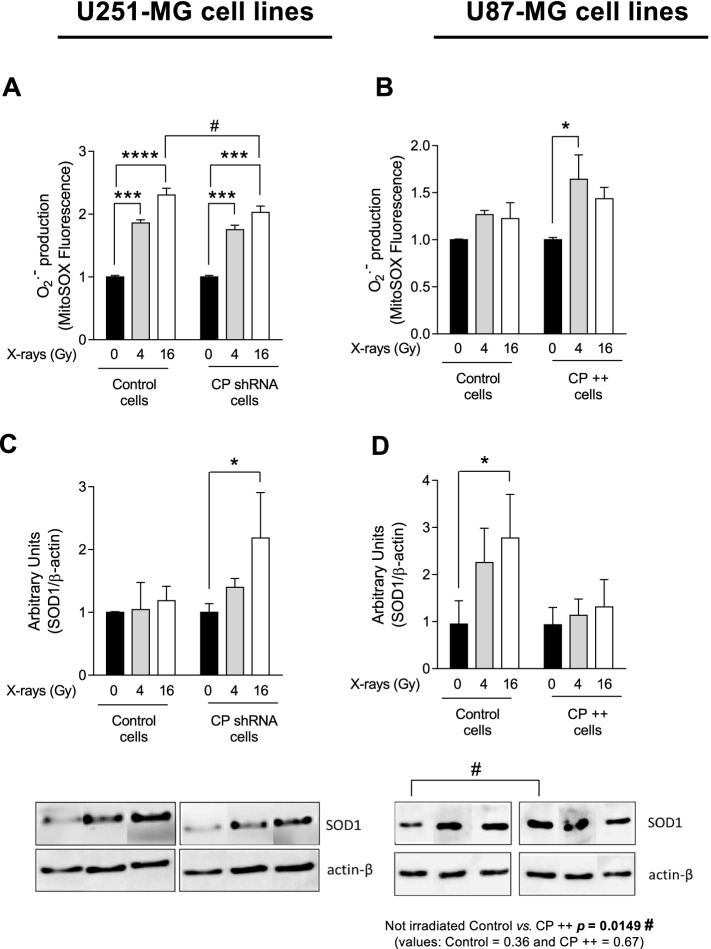
Change in superoxide anion production and SOD1 expression in CP-modulated cells post-IR. Superoxide anion production was evaluated by flow cytometry measuring MitoSOX™ fluorescence in stable transfected U251-MG (**A**) and U87-MG GB (**B**) cell lines 24 h after a single exposure to 4 Gy- or 16 Gy-irradiation at 3% oxygen. Anion superoxide is expressed as % of positive cells ± SEM (*n* = 3). In same conditions, western blotting was performed to evaluate SOD1 expression in U251-MG (**C**) and U87-MG cells (**D**). SOD1 expression is expressed as mean ± SEM (*n* = 4). One-way ANOVA with multiple comparisons (each group was compared to their own normalized control) was performed between Control vs. irradiated cells (^***^, *p*-value ≤ 0.001; ^****^, *p*-value ≤ 0.0001). Two-way ANOVA was performed when cell populations have been compared (#, *p*-value ≤ 0.05)

In order to measure superoxide anion O_2_^**.**−^ production, a MitoSOX™ red mitochondrial fluorescent indicator was used and evaluated by flow cytometry 24 h after an exposure to irradiation in CP shRNA cells and in CP +  + cells. Results showed a significant increase of O_2_^**.**−^ production after 4 and 16 Gy-irradiation in both U251-MG cell lines and to a loer extent in both U87-MG cell lines (less sensitive) (Figs. 5A and 5B). However, the increase in oxidation of the MitoSOX™ red reagent by superoxide anion producing red fluorescence appears less marked in U251-MG cells down regulated for CP at 16 Gy (Fig. 5A) and more marked in U87-MG cells overexpressing CP at 4 and 16 Gy (Fig. 5B).

The expression of superoxide dismutase 1 (SOD1; Cu–Zn SOD), involved in oxidative regulation, was also investigated in these cells. As expected, the irradiation induces the expression of SOD1 in a dose-dependent manner in both U251-MG and U87-MG cells (Fig. 5C and D). However, in non-irradiated cells, depletion of CP in U251-MG cells resulted in moderate or no impact on SOD1 expression (Fig. 5C), while overexpression of CP in U87-MG cells induces SOD1 to a maximal level (significantly different with *p* = 0.0149 compared to control) that is not further modified by irradiation (CP +  + cells, Fig. 5D).

This latter observation supports the possibility that in the presence of an elevated level of CP, GB intracellular detoxification mechanisms may be more necessary (basal level of SOD1 expression to be maximum even in the absence of radiations).

### Impact of CP on DNA damage and repair capacity following ionizing radiation

**Fig. 6:**
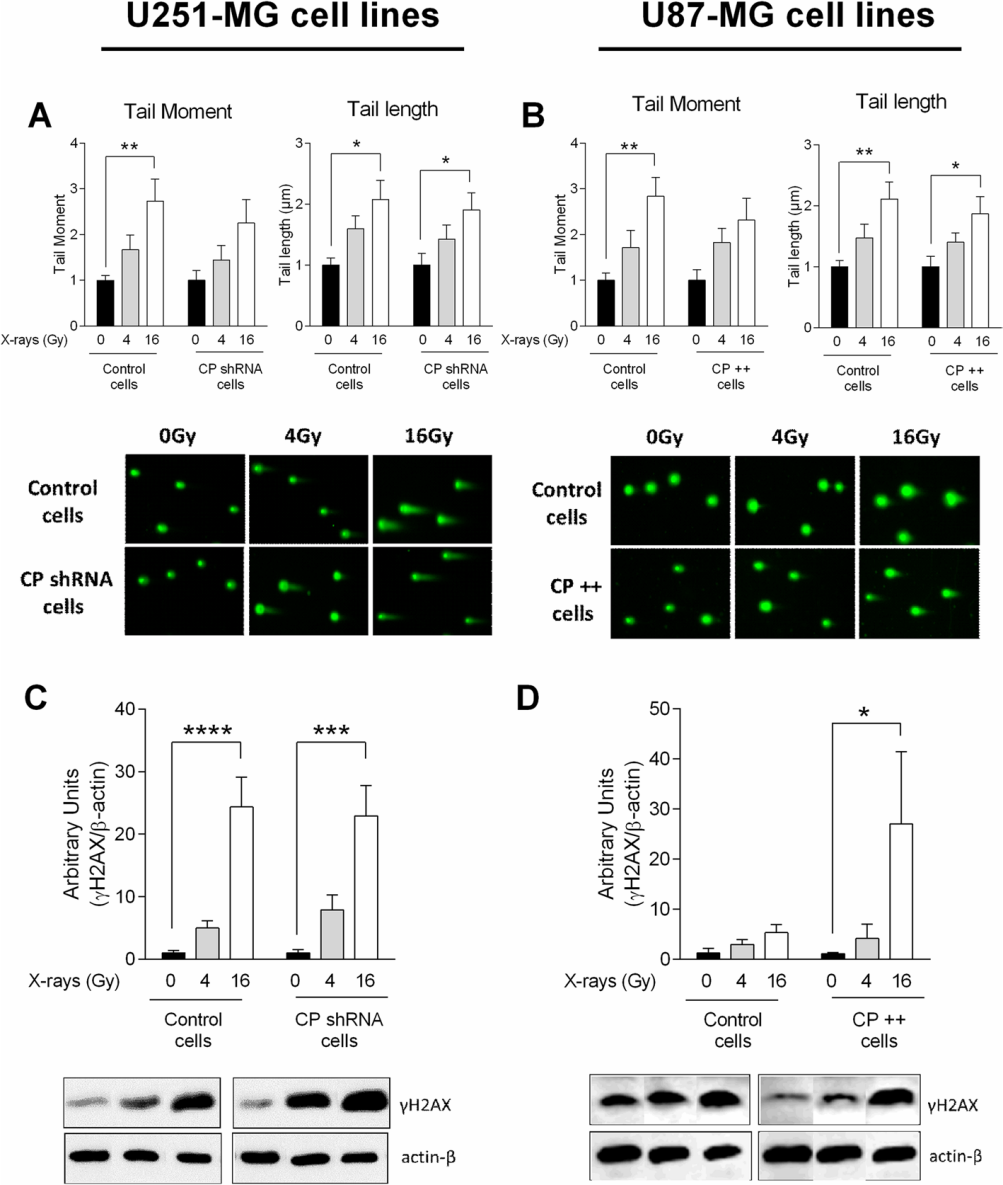
DNA damage and expression of DNA repair marker in CP-modulated cells post-IR. Single cell electrophoresis comet assay was performed to measure DNA double-strand breaks in stable transfected U251-MG (**A**) and U87-MG GB (**B**) cell lines 2 h after a single exposure to 4 Gy- or 16 Gy-irradiation at 3% oxygen. Tail moment, corresponding to %DNA in tail multiplied by length of tail, is represented in left graph while length of tail is represented in right graph (**A** and **B**). Results are expressed as mean ± SEM (*n* = 7). Then, in same condition, western blotting was performed to evaluate γH2AX expression in U251-MG (**C**) and U87-MG cells (**D**) and results are expressed as mean ± SEM (*n* = 4–7). One-way ANOVA with multiple comparisons (each group was compared to their own normalized control) was performed between Control vs. irradiated cells (^*^, *p*-value ≤ 0.05; ^**^, *p*-value ≤ 0.01; ^***^, *p*-value ≤ 0.001; ^****^, *p*-value ≤ 0.0001)

The response of cancer cells to irradiation is notably linked to DNA damage together with repair capacity. To evaluate the DNA damage response, single cell electrophoresis comet assay was performed at two hours after exposure to 0, 4 and 16 Gy-irradiation in CP-depleted U251-MG and in CP-overexpressing U87-MG cells. Phosphorylation of the Ser-139 residue of the histone variant H2AX, forming γH2AX, as a sensitive molecular marker of DNA damage and repair was conjointly evaluated at two hours. Tail moment corresponding to % of DNA in tail multiplied by length of tail (left panel) and length of tail (right panel) were quantified (Figs. 6A and B). Although a significant increase of comet’s tail moment and tail length occurred after irradiation in all cell lines, modification of CP expression did not result in any change toward direct DNA damages evaluated through this assay (Figs. 6A and B). In contrast, while a significant increase of γH2AX was induced by 4 and 16 Gy irradiation in all cell types (Figs. 6C and D), a strong up-regulation of this marker was observed at 16 Gy due to CP overexpression (U87-MG CP +  + cells compared with their Control cells, Fig. 6D). This last result is likely supportive of an increased need for repair or increase cell damage due to the overexpression of CP in the presence of an irradiation.

### Impact of GB cell CP status on intracellular iron levels in response to irradiation

**Fig. 7:**
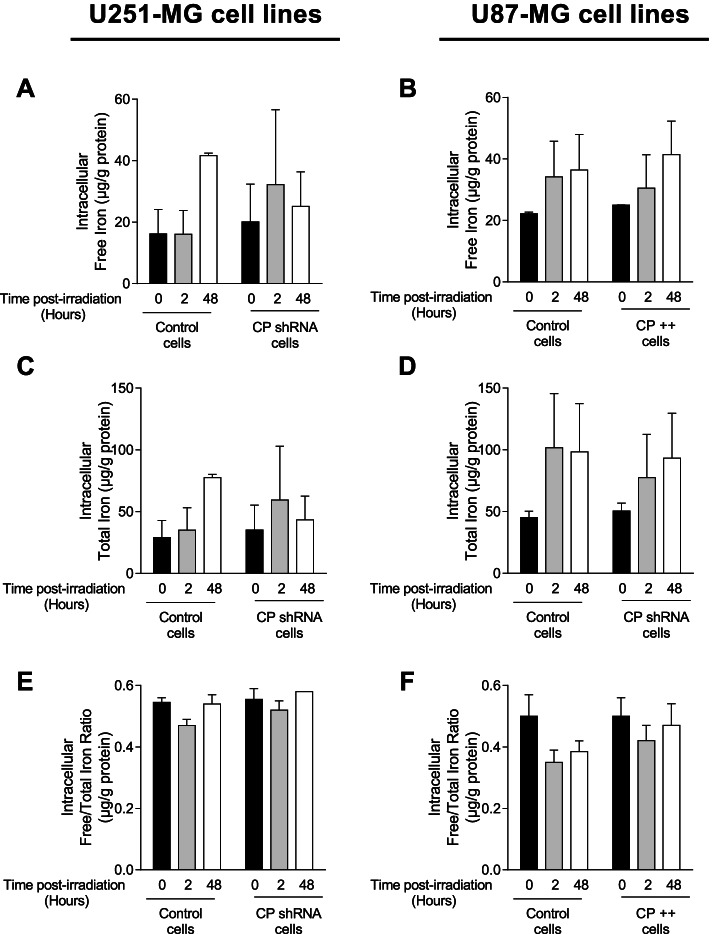
Intracellular iron in CP-modulated cells post-IR. Intracellular free iron (**A** and **B**), intracellular total iron (**C** and **D**) and free/total iron ratio (**E** and **F**) were measured, using ICP-MS, in stable transfected U251-MG (**A, C and E**) and U87-MG GB (**B, D** and **F**) cell lines 2 and 48 h after a single exposure to 16 Gy-irradiation at 3% oxygen. Iron is expressed in µg/g of protein and as mean ± SEM (*n* = 2)

As iron collaborates with oxygen, in Fenton’s reaction to produce ROS then partly responsible for DNA damage after exposure to radiations and as CP plays a role in cells capacity to load iron, the impact of irradiation on the amount of intracellular free, bound and total iron depending on the CP status of GB cells was determined (Fig. 7). 2 and 48 h after 16 Gy-irradiation, total iron that include intracellular bound and free iron is upregulated in both control U251-MG (Fig. 7A, C and E) and U87-MG cells (Fig. 7B, D and F). In contrast, 48 h after irradiation in CP shRNA cells, iron levels are not induced to the same extent as when CP is expressed basally and not down regulated (Fig. 7A, C and E). In addition, upregulation of CP in U87-MG cells (CP +  + cells) had no impact on iron levels following irradiation.

## Discussion

**Fig. 8:**
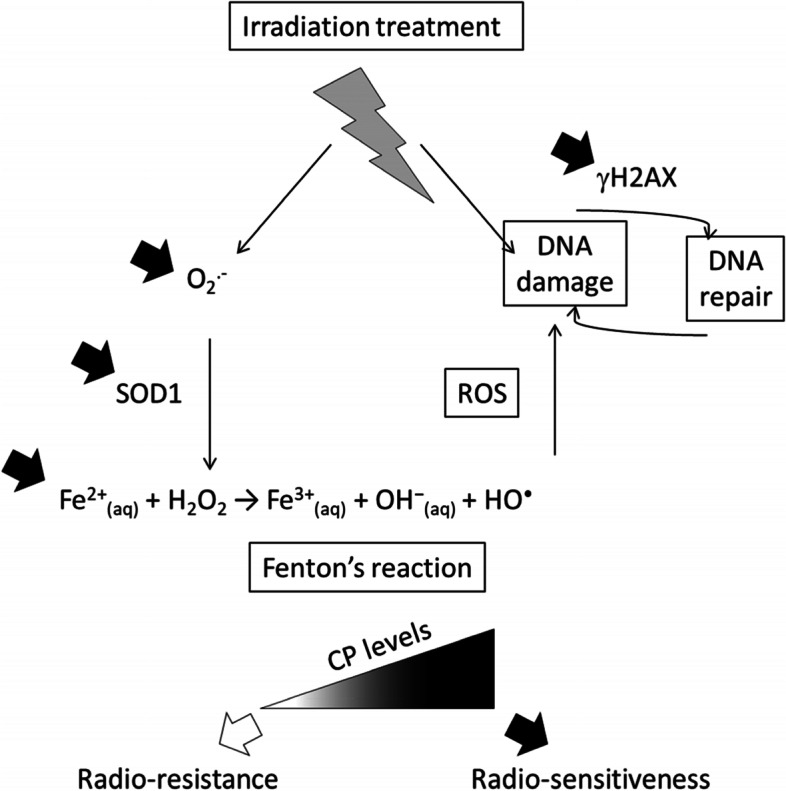
CP as an intracellular potentiator of radiation responses in GB cells. The manipulation of CP expression in GB lines impacts both cell survival and activation of DNA repair/damage machinery (γH2AX), together with the production of reactive oxygen species with more superoxide anion, more SOD1 synthesis and more cellular Fe^2+^ when intracellular CP is present at high levels. These observations reemphasized also the role of Fenton's reaction tightly involved in the production of ROS by GB cells which might be one of the major link between intracellular CP levels and GB cell responses/survival to radiation. Hence, “GB prepared cells” under-expressing CP and “GB adapter cells” that naturally down regulate CP survive better to radiations thus providing a role for CP in the radio-resistance/radio-sensitiveness balance of GB cells

GB are very aggressive tumors and are particularly resistant to treatments that include irradiation as a first line arsenal. Although several biological mechanisms are put forward to explain the phenomenon promoting escape, prevention and repair, including tumor hypoxia, GB stem-like cell status, GB genomic instability and occurrence of resistant clones [33], a greater understanding the molecular and cellular events which occurs during radioresistance is an essential objective in the management of GB. Amongst factors within GB cells that can be considered as ways to revolutionize the therapeutic approach, iron signaling remains to be elucidated, especially in light of what has been learned about the radio-resistant hypoxic environment. In this context, the present study was carried out by trying to define if iron has a role in GB cell resistance to radiation therapy, suggesting a possible involvement for iron regulatory protein partners. Hence, among the protein partners of iron tested the only one regulated at the transcriptional level by irradiation was found to be CP, then under-expressed in “GB prepared cells” or downregulated in “GB adapter cells” surviving to the treatment. Witness of suffering or actor of the effects of irradiation, manipulation of CP in two GB cell lines was then tested, thus demonstrating in turn that U87-MG CP +  + cells are less viable after irradiation than their control counterpart and that U251-MG CP shRNA cells die less than control cells by apoptosis in response to radiation. It was observed that U87-MG CP +  + cells produce more ROS and have more SOD1, as well as increased γH2AX levels in response to irradiation. Furthermore, the reduction of CP (U251-MG CP shRNA cells) limits the upregulation of intracellular iron levels induced by irradiation. All of these data emphasize a role for CP in sensitizing the molecular responses of GB cells to radiation. They also support the concept that downregulation or low intracellular levels of CP represents a mechanism of resistance to irradiation treatment of GB cells prepared for it, in particular with regard to iron metabolism and Fenton's reaction (Fig. 8).

Living organisms, including cancer cells have had to adapt to iron, one of the most abundant and ancient elements involved in oxygen metabolism. In this respect, the effects of pO_2_ and hypoxia on radiation resistance are beginning to be well documented, although it is still difficult to therapeutically manipulate them [21]. The pivotal role that iron could play in tumor survival has emerged in the last decade. On the one hand, iron plays a key role in many biologic processes, such as transport of molecular oxygen, heme biosynthesis, DNA synthesis or electron transfer [24]. On the other hand, the phenomenon of “iron addiction” of cancer cells has been highlighted, indeed cancer cells need more iron than healthy cells to proliferate and play a role in tumor growth [4]. The use of iron chelators has proved its effectiveness in clinical studies on diseases other than cancer [6]. In the context of GB, our team highlighted the benefit effect of deferasirox, to reduce intracellular iron overload, on GB cell lines but only in normoxia since it has no effect under hypoxia. GB has many hypoxic areas which shows the complexity of using iron chelators as anticancer therapy [19]. Thereby, the role of iron metabolism in GB therapy is an attractive track that must be reappraised in view of its intracellular deleterious functions that could be helpful to promote tumor cell death notably in response to radiation treatments.

### Involvement of CP in the iron metabolism of GB cell in response to radiations

The choice was made to work with two cell lines responding differently to radiotherapy with U251-MG being radiosensitive while U87-MG is radioresistant as shown in this study but also described in the literature [31]. It is known that GB cell lines with mutant gene TP53, as is the case for U251-MG, are more sensitive to radiation. U87-MG cells present wilt-type TP53 gene which enhances resistance of these cells to chemotherapy, such as temozolomide [34]. In addition, Hsieh et al. has shown that cycling hypoxia causes radioresistance of U87-MG glioma cells through ROS induced HIF-1 signal transduction activity [35]. Once again, hypoxia seems to play a central role in the radioresistance phenomenon. Because of this, hypoxic conditions (3% O_2_) were used for the entire remaining study. Iron intake and intracellular distribution must be strictly regulated, which is achieved by a set of specific proteins involved in iron metabolism [36]. A first study was performed to evaluate profile expression of cellular iron metabolism partners following external irradiation. In our study, among studied genes involved in iron uptake (TfR1 and DMT1), storage (Ferritin) or in mitochondria metabolism (mitoferrin and frataxin), only one gene involved in iron efflux the CP (not FPN1) is downregulated at day 8 after IR, as at the protein level, in both radiosensitive and radioresistant cell type and under hypoxia.

Although all functions of this protein remain to be documented, the first major roles described in the literature were as a copper transporter, then in iron homeostasis, with its ferroxidase activity, converting ferrous ions [Fe^2+^] into ferric ions [Fe^3+^] in the extracellular compartment [26, 28]. Interestingly, CP is involved in both iron influx and efflux, respectively allowing capture of ferric ions by transferrin for the transport into the cell, but also facilitating the export activity of ferroportin, with a molecular connection between both proteins, required for iron efflux out of the cell [37, 38]. It has been shown in rat astrocytes that CP is predominantly expressed in a form anchored to the membrane with a glycophosphatidylinositol (GPI) and that this form is necessary for the efflux of iron [39] while another secreted form is predominant in hepatocytes [40]. These two forms, anchored and secreted, come from the same gene which is subjected to alternative splicing [32]. The importance of CP in brain iron metabolism has been demonstrated in patients with the genetic disease aceruloplasminemia. The lack of activity of this ferroxidase leads to iron overload in various organs, including liver and brain, resulting in neurological disorders in adults [41, 42]. Referring to the role of iron in GB cells [23] and in particular stem-type cancer cells [10, 43], but also at the level of the dynamics of the tumor ecosystem taken as a whole, then the role of CP is undoubtedly much more complex than initially thought. As such, CP is both intrinsically (e.g. RNA interference) and extrinsically regulated (e.g. extracellular matrix) and is involved in a myriad of regulatory mechanisms (e.g. HIF stabilization, angiogenesis, etc.) [44]. Hence, while hyaluronan has been shown to regulate CP production in GB and their treatment-resistant multipotent progenitors, no correlation so far was demonstrated between CP and GB cells radioresistance or radiosensitivity [45]. Hence, the current in vitro study represents a first at the cellular intrinsic level.

### Involvement of CP in ROS production and GB cellular damage in response to radiation

Based on our findings, we hypothesize that CP downregulation after irradiation either i) reflects cellular stress or, alternatively, ii) enables GB cells to better resist radiation. Interestingly, a recent study showed reduced CP in whole blood of patients with breast cancer after delivery of radiation [46]. Since radiotherapy aims to eradicate tumor by inducing cancer cell death, its potential role could be assigned to different functions such as: i) prevention of damage by deregulating the cycle of iron and therefore its cellular availability and by extension its contribution in the Fenton reaction and ROS production, or ii) a direct link with DNA damage or increase the capacity of repair machinery. The present study was aimed at evaluating the specific role of CP in irradiation efficiency in GB cells. For this, CP expression was modulated in U251-MG and U87-MG cells. A downregulation of CP protein and gene was confirmed in U251-MG radiosensitive cell line while an upregulation was confirmed in U87-MG radioresistant cell line. Nevertheless, our results seem to show a disconnect between CP protein expression and levels of mRNA, suggesting a role for post-transcriptional regulation for CP, as already described for other iron partners such as TfR1 and ferritin [47].

In order to determine if CP modulation expression impacts cells viability in response to radiation, MTS assay and late and early apoptosis were measured in parallel. This revealed a lower radiosensitivity when CP was depleted in cells or vice versa, although these effects seem moderated. This suggests a positive role for CP in the mechanisms that lead to death, such as toxic signals generated by radiations (ROS production, DNA damage…) or, conversely, an inhibitory, regulatory role in the mechanisms that lead to survival or repair. Since CP seems to influence cells outcome, viability and death, following irradiation, it was necessary to evaluate associated mechanisms such as oxidative stress and DNA damage/repair.

Oxidative stress investigations indicated that U251-MG CP-depleted cells, in contrast to U87-MG CP-overexpressing cells, produce slightly less superoxide anions and express more SOD1 after exposure to radiation. These data supported our previous observations demonstrating less apoptosis in these cells and lower RX irradiation efficacy when CP is decreased. DNA damage was not affected by variation in CP expression with no change in the induction of DNA breaks (direct) at early time after exposure (2 h), though this does not exclude potential indirect impact (e.g., through ROS) at a later time. In line with this, the DNA repair marker γ-H2AX was increased only when CP was overexpressed in U87-MG cells line, an observation that could reflect an adaptation in response to greater stress of these cells.

Due to its ferroxidase activity, CP has a role in the management of oxidative stress, though depending on the context, this can be either pro-oxidant or anti-oxidant molecule [48]. In fact, by its oxidase activity, CP allows iron and copper to be trapped and thus prevent the Fenton reaction, thus limiting their deleterious pro-oxidant effect [29]. Indeed, Fe (II), via the Fenton reaction, leads to the production of ROS, while Fe (III) is very quickly sequestered by transferrin. CP also has an inherent ROS scavenger function [49, 50]. Very recently, Tian et al. highlighted that a tyrosine residue in CP prevents ROS formation when Fe^2+^ delivery is dysregulated [51]. Due to its antioxidant properties, it prevents the formation of ROS in the CNS acting as a protector against neurotoxicity induced by kainate or traumatic brain injury and also protects against oxidative damage in pancreas by facilitating iron efflux [52–55]. Importantly, these functions are enhanced when the CP is bonded with copper [56]. In the same way, depletion of CP resulted in accumulation of ROS in astrocytes [57] but also in hepatocellular carcinoma cells, thus in a tumor context [58]. In a contradictory way, CP itself may have pro-oxidant activity leading to DNA damage induced by hydrogen peroxide or releasing copper ions generating protein carbonyl derivatives [59, 60]. In present study, we highlighted a pro-oxidant effect of CP following irradiation. Interestingly, Boyd et. al, has demonstrated that the antioxidant SOD1 requires copper to scavenge O_2_^**.−** ^[61]. So, in this case, depletion of CP would increase copper bioavailability to activate SOD1 and consequently decrease O_2_^**.−**^ production.

Conversely, several studies demonstrate the involvement of redox copper in the CP regulation with, for example, AP-1 site in the CP gene [62]. This regulation by redox copper may also explain previous findings of increased CP expression in several cancers, where the intracellular copper level is higher in a redox compromised environment, such as in GB [63].

The impact of CP knockdown at the protein level is weaker than at the transcript level, thus demonstrating an important translational/post-translational regulation of this protein. Overall, these results attest that slight variation of its expression result in a moderate but significant role for CP in GB cell. The moderate response could be explained by a compensation with the CP homologue, the hephaestin, ferroxidase, also converting ferrous iron to ferric iron for the transport of dietary iron in the circulatory system [27]. Because hephaestin is a membrane-bound ferroxidase that partners with FPN1 to facilitate iron efflux and was reported to be expressed in human brain astrocytes and in mouse brain, it may partially compensate for loss of the GPI-CP form [39, 64, 65].

Beyond iron metabolism, the superoxide radical plays a major role in the production of ROS, the impact of which on cell survival is preponderant. In this regard, SOD1 constitutes the key factor catalyzing the disproportionation of O2^.−^. If CP is capable of such activity without formation of hydrogen peroxide, this is reduced by at least a factor of 100 [66]. Stressed tumor cells therefore have a real interest for their survival in using the key enzyme, SOD1. However, it was found that the stability of the activity of SOD1 and that of CP with regard to radiation is not equal and in favor of CP [67]. Our observations under irradiation demonstrating an increased quantity of O2^.−^ and an over-synthesis of SOD1 in the presence of more CP go in this direction of a return to an effective SOD1 detoxification in favor of the less useful CP. The initial downregulation of intracellular CP following irradiation therefore possibly has the double effect of mobilizing less iron in its deleterious part for the cells (Fenton reaction) and of better detoxifying the cell with regard to reactive oxygen species. To this end, the overproduction of γH2AX also points in this direction to an increased stress / need for increased repair of GB tumor cells in response to radiation in the presence of more CP. All of these concepts are summarized in Fig. 8.

The effects that we have observed on the modulation of CP, cell survival, DNA damage/repair and on the production of ROS, have been investigated under a single RX irradiation modality. Hence, applying dose fractionation, vectorized targeted irradiation using a beta or alpha radionuclide or even flash therapy may result in revealing further the role of CP highlighted in this study notably in the in vivo context. These experimental data should therefore be considered with CP as partner of radio-efficiency and/or as an intracellular witness of radio-sensitiveness. A recent study showed that reduced expression of FPN1 and CP in adrenocortical carcinoma was associated with poor prognosis, thus supporting these proteins as a potential biomarkers of cancer signature and treatment responses [68].

In conclusion, our in vitro results demonstrate for the first time that CP plays a positive role in the efficiency of radiotherapy on GB cells and underlie the idea that radiotherapies may have to be adapted, when necessary, to the CP profile (or status) of tumor cells. In this regard, cellular CP could be regarded as a biomarker indicating but also promoting the effectiveness of therapeutic doses of radiation. Also, CP down regulation by irradiation in GB cells could be seen as a GB cell protective mechanism ascribed to prevention, repair and expulsion of unwanted compounds for survival.

## Supplementary Information

**Additional file 1.** Original, unprocessed versions of westernblot.

## Data Availability

The datasets used and/or analyzed during the current study available from the corresponding author on reasonable request.

## Abbreviations

CP: Ceruloplasmin
DNA: DeoxyriboNucleic Acid
DMEM: Dulbecco’s modified Eagle's medium
DMT1: Divalent metal transporter-1
FBS: Fetal bovine serum
FPN1: Ferroportin
FTH1: Ferritin Heavy Chain
FTL: Ferritin Light Chain
FXN: Frataxin
GB: Glioblastoma
GPI: Glycosylphosphatidylinositol
Gy: Grays
LDH: Lactate DeHydrogenase
MFRN1, 2: Mitoferrin 1 and 2
ROS: Reactive oxygen species
TF: Transferrin
TFR1: Transferrin Receptor 1
