# Supplementary material for: A role for ceruloplasmin in the control of human glioblastoma cell responses to radiation
Source: BMC Cancer. 2022 Aug 2;22:843. doi: 10.1186/s12885-022-09808-6 (PMC9347084; doi:10.1186/s12885-022-09808-6)

**Supplementary Information file**

Original, unprocessed versions of western blot :

1. Western blot SOD1 in U251-MG cell-lines ; Figure 5C in manuscript


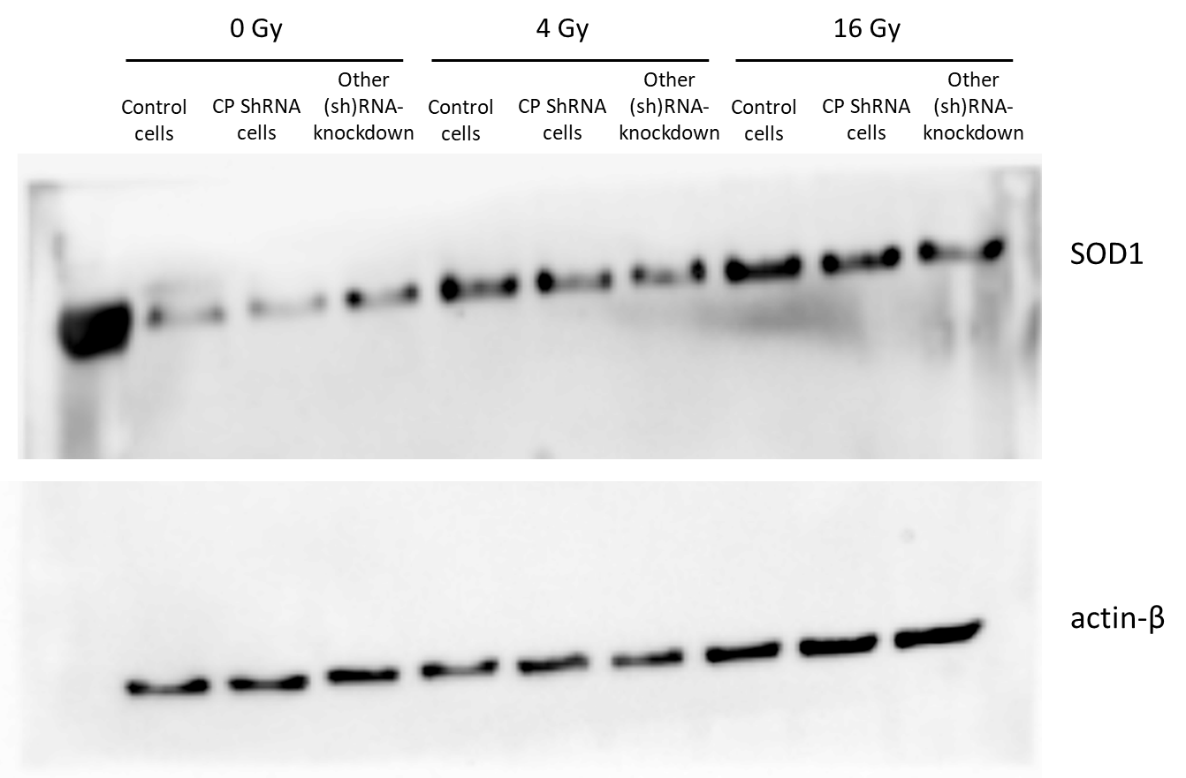


1. Western blot SOD1 in U87-MG cell-lines ; Figure 5D in manuscript


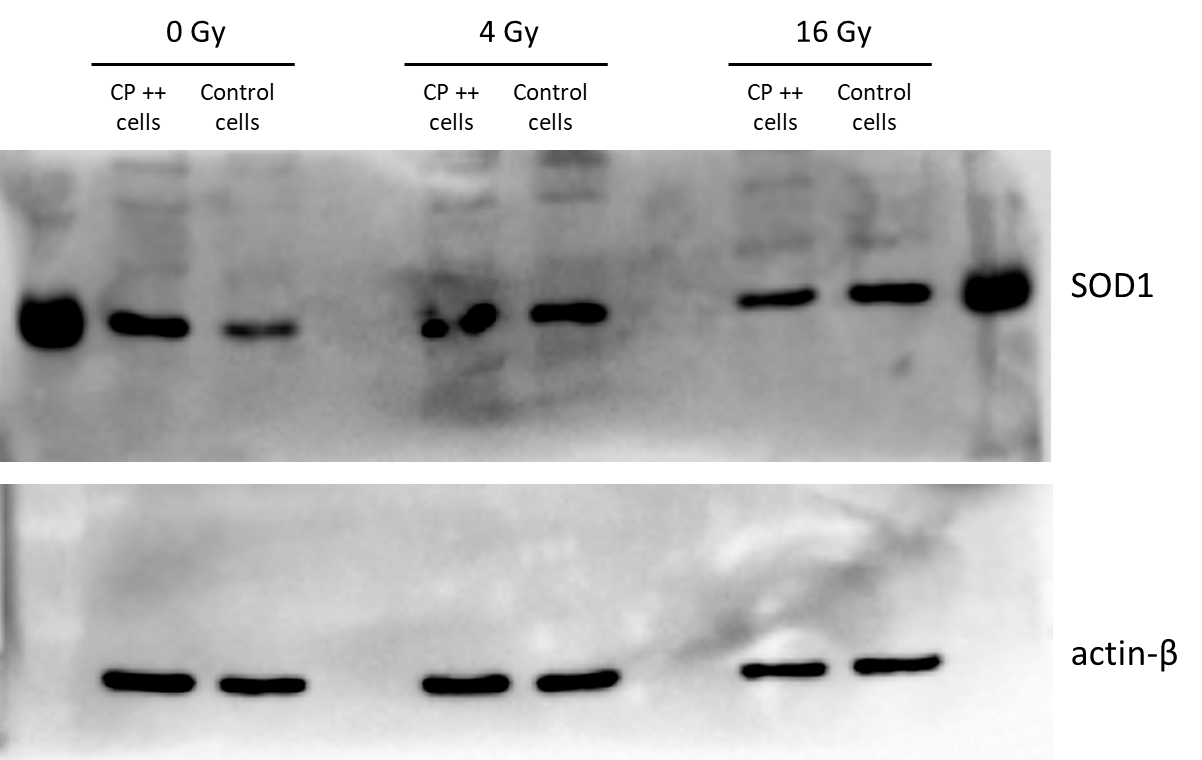


1. Western blot gH2AX in U251-MG cell-lines ; Figure 5C in manuscript


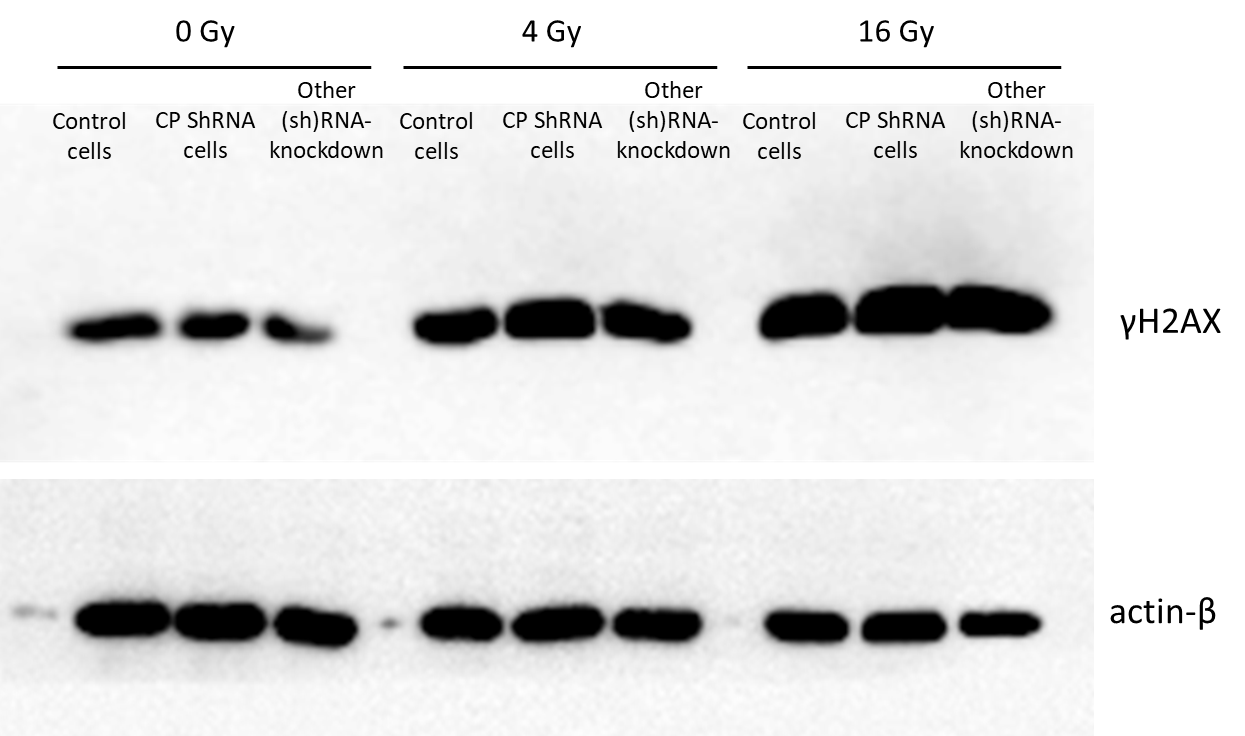


1. Western blot gH2AX in U87-MG cell-lines ; Figure 5D in manuscript


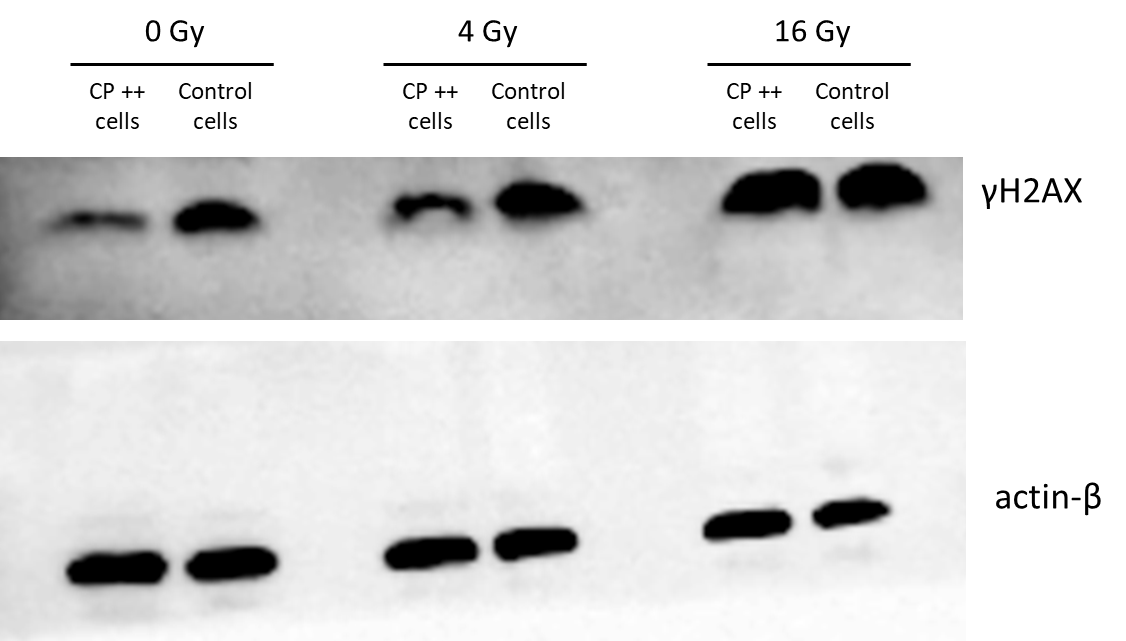

Supplement: Supplementary file 1 — Additional file 1. Original, unprocessed versions of westernblot. [file 12885_2022_9808_MOESM1_ESM.docx]
